# Effect of Leguminous Lectins on the Growth of *Rhizobium tropici* CIAT899

**DOI:** 10.3390/molecules18055792

**Published:** 2013-05-17

**Authors:** Mayron Alves de Vasconcelos, Cláudio Oliveira Cunha, Francisco Vassiliepe Sousa Arruda, Victor Alves Carneiro, Rafaela Mesquita Bastos, Fábio Martins Mercante, Kyria Santiago do Nascimento, Benildo Sousa Cavada, Ricardo Pires dos Santos, Edson Holanda Teixeira

**Affiliations:** 1 Department of Biochemistry and Molecular Biology, Federal University of Ceará, Fortaleza, CE, 60440-970, Brazil; E-Mails: mayronvasconcelos@gmail.com (M.A.V.); agrobrasil@gmail.com (C.O.C.); kyriasantiago@ufc.br (K.S.N.); bscavada@gmail.com (B.S.C.); 2 Integrated Laboratory of Biomolecules (LIBS), Department of Pathology and Legal Medicine, Faculty of Medicine, Federal University of Ceará, Fortaleza, CE, 60430-160, Brazil; E-Mails: vassiliepe@gmail.com (F.V.S.A.); victorcarneiro@ufc.br (V.A.C.); rafaelabastos.ufc@gmail.com (R.M.B.); 3 Embrapa Western Region Agriculture, Dourados, MS, 79804-970, Brazil; E-Mail: fabio.mercante@embrapa.br; 4 Laboratory of Materials Engineering and Computation of Sobral (LEMCS), Federal University of Ceará, Campus do Derby, Sobral, CE, 62042-280, Brazil; E-Mail: rpsantos2007@gmail.com

**Keywords:** rhizobia, *Phaseolus vulgaris*, stimulus, inoculant additive

## Abstract

*Rhizobium tropici* is a Gram-negative bacterium that induces nodules and fixed atmospheric nitrogen in symbiotic association with *Phaseolus vulgaris* (common bean) and some other leguminous species. Lectins are proteins that specifically bind to carbohydrates and, consequently, modulate different biological functions. In this study, the d-glucose/d-mannose-binding lectins (from seeds of *Dioclea megacarpa*, *D. rostrata* and *D. violacea*) and d-galactose-binding lectins (from seeds of *Bauhinia variegata*, *Erythina velutina* and *Vatairea macrocarpa*) were purified using chromatographic techniques and evaluated for their effect on the growth of *R. tropici* CIAT899. All lectins were assayed with a satisfactory degree of purity according to SDS-PAGE analysis, and stimulated bacterial growth; in particular, the *Dioclea rostrata* lectin was the most active among all tested proteins. As confirmed in the present study, both d-galactose- and d-glucose/d-mannose-binding lectins purified from the seeds of leguminous plants may be powerful biotechnological tools to stimulate the growth of *R. tropici* CIAT99, thus improving symbiotic interaction between rhizobia and common bean and, hence, the production of this field crop.

## 1. Introduction

Rhizobia are soil bacteria best known as root-nodule symbionts of legumes [1]. In 1991, Martinez-Romero and colleagues [2] identified a new species of common bean symbionts: *Rhizobium tropici*. This new rhizobial species was described as aerobic, Gram-negative, with optimal pH for growth ranging between 5 and 7, and characterized by high genetic stability of the symbiotic plasmid and tolerance to tropical environmental stresses such as high temperature and low soil pH [2]. Strain CIAT899, known commercially as SEMIA 4077, the type-strain of *R. tropici*, was isolated at the Centro Internacional de Agricultura Tropical (CIAT) in Colombia, and it was recognized as an effective symbiont of the common bean, *Leucaena leucocephala* and some other leguminous species [2,3].

Some studies have established the role of *R. tropici* in competition with other rhizobia during successive bean cultures and *R. tropici* was judged more competitive than either *Rhizobium leguminosarum* or *Rhizobium etli* [4,5]. Moreover, based on its superior characteristics as a common bean root-nodule symbiont, this strain is currently recommended (authorized) for the production of commercial rhizobial inoculant for common bean production in Brazil [6].

Lectins are proteins of nonimmune origin that bind to specific carbohydrates without modifying them. They are organized into structurally closely related families [7]. Because of their carbohydrate recognition ability, lectins are involved in important cellular events, such as symbiotic and pathogenic interaction between microorganisms and hosts [8]. In fact, lectins actively participate in the biological events involving the symbiosis between rhizobia and host plant [9,10]. These proteins mediate the attachment of rhizobia cells in the roots [9], agglutination of rhizobia near the roots [11] and recognition of nodulation factors [12,13]. Some studies showed that lectins may interact with rhizobia and modulate its metabolism, inducing the protein synthesis [14], increasing cellular respiration [15], stimulating the efflux of H^+^ [16] and promoting bacterial growth [17,18]. Moreover, the use of lectins can enhance the symbiotic efficiency between rhizobia and host plant, leading to an improvement in plant productivity [19,20].

The aim of this study was to evaluate the effect of three different d-glucose/d-mannose-specific lectins (purified from seeds of *Dioclea megacarpa*, *D. rostrata* and *D. violacea*) and three d-galactose specific lectins (purified from seeds of *Bauhinia variegata*, *Erythina velutina* and *Vatairea macrocarpa*) on the *in vitro* growth of *R. tropici* CIAT 899.

## 2. Results and Discussion

**Figure 1:**
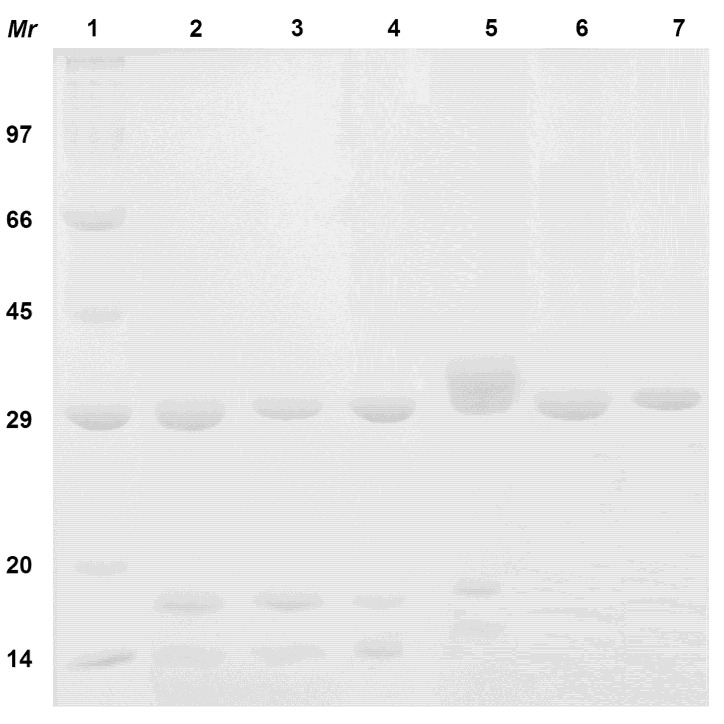
SDS-PAGE of the purified lectins. (1) Molecular mass markers: phosphorylase b, 97 kDa; bovine serum albumin, 66 kDa; ovalbumin, 45 kDa; carbonic anhydrase, 29 kDa; trypsin inhibitor, 20.1 kDa; α-lactalbumin, 14.4, (2) *D. rostrata* lectin, (3) *D. megacarpa* lectin, (4) *D. violacea* lectin, (5) *V. macrocarpa* lectin, (6) *E. velutina* lectin and (7) *B. variegata* lectin.

The SDS-PAGE of *D*. *megacarpa* lectin (DML), *D. rostrata* lectin (DRL) and *D. violacea* (DVL) showed a pattern of subunits characteristic of lectins from the Diocleinae subtribe. The proteins migrated as three bands consisting of the full-length intact polypeptide chain (α-chain) and two fragments, β and γ (Figure 1). *V*. *macrocarpa* lectin (VML) showed a four-band pattern, consisting of two major bands, termed α-chains (34 and 32 kDa), and two minors bands, termed β- and γ-chains (22 and 13 kDa, respectively). A different pattern was seen for *B*. *variegata* lectin (BVL) and *E*. *velutina* lectin (EVL), which showed only one band for each protein of approximately 32 kDa (Figure 1). Moreover, all lectins showed hemagglutinating activity against rabbit erythrocytes and were fully inhibited by 0.1 M D-mannose (DML, DRL and DVL) and 0.1 M D-galactose (BVL, EVL and VML) (data not shown).

**Figure 2:**
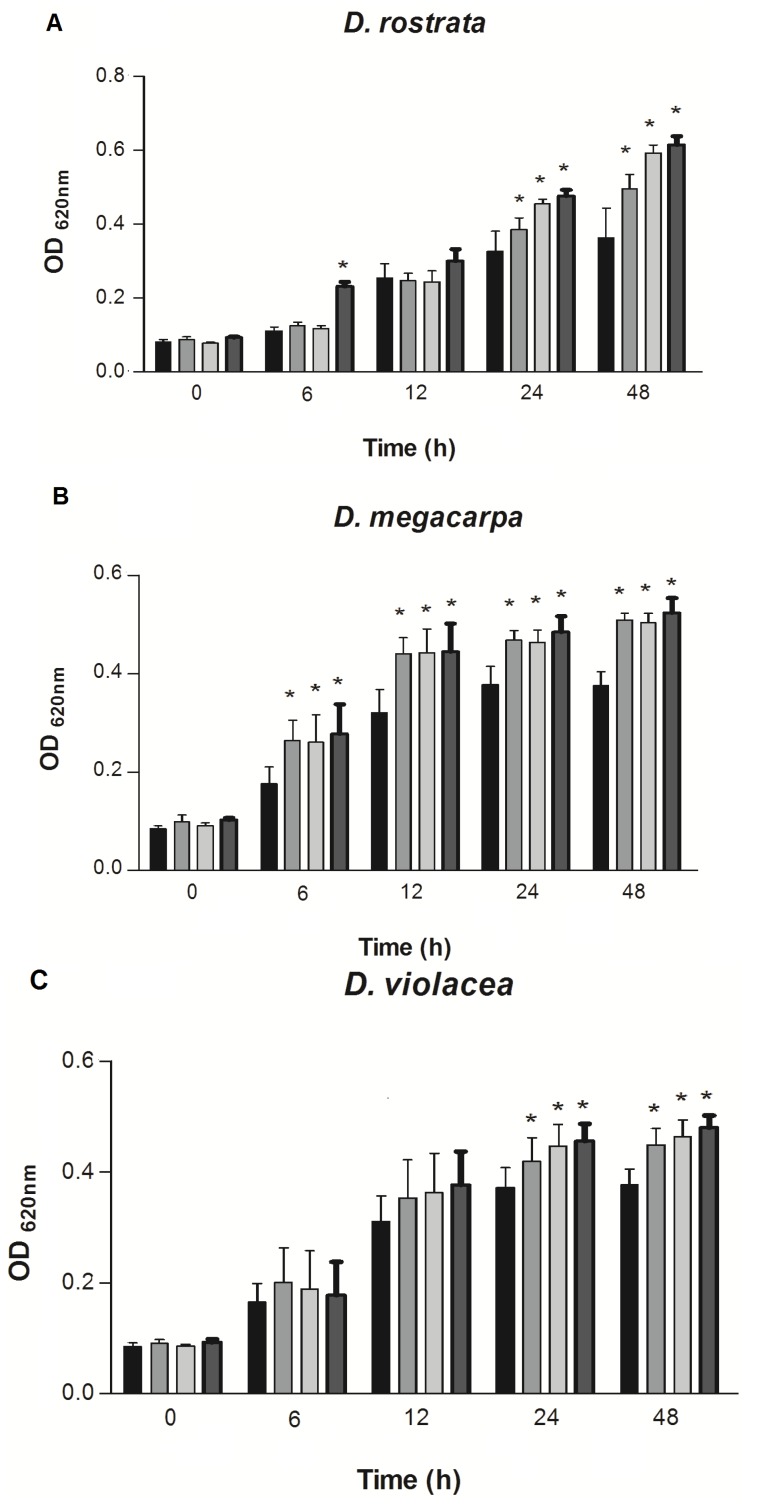
Effect of d-glucose/d-mannose-specific lectins on the growth of *R. tropici*. (**A**) DRL, (**B**) DML and (**C**) DVL. * *p* < 0.01 relative to control. Error bars indicate the standard deviation of the means. (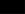) Control, (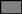) lectins at 125 µg/mL, (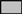) 250 µg/mL and (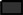) 500 µg/mL.

**Figure 3:**
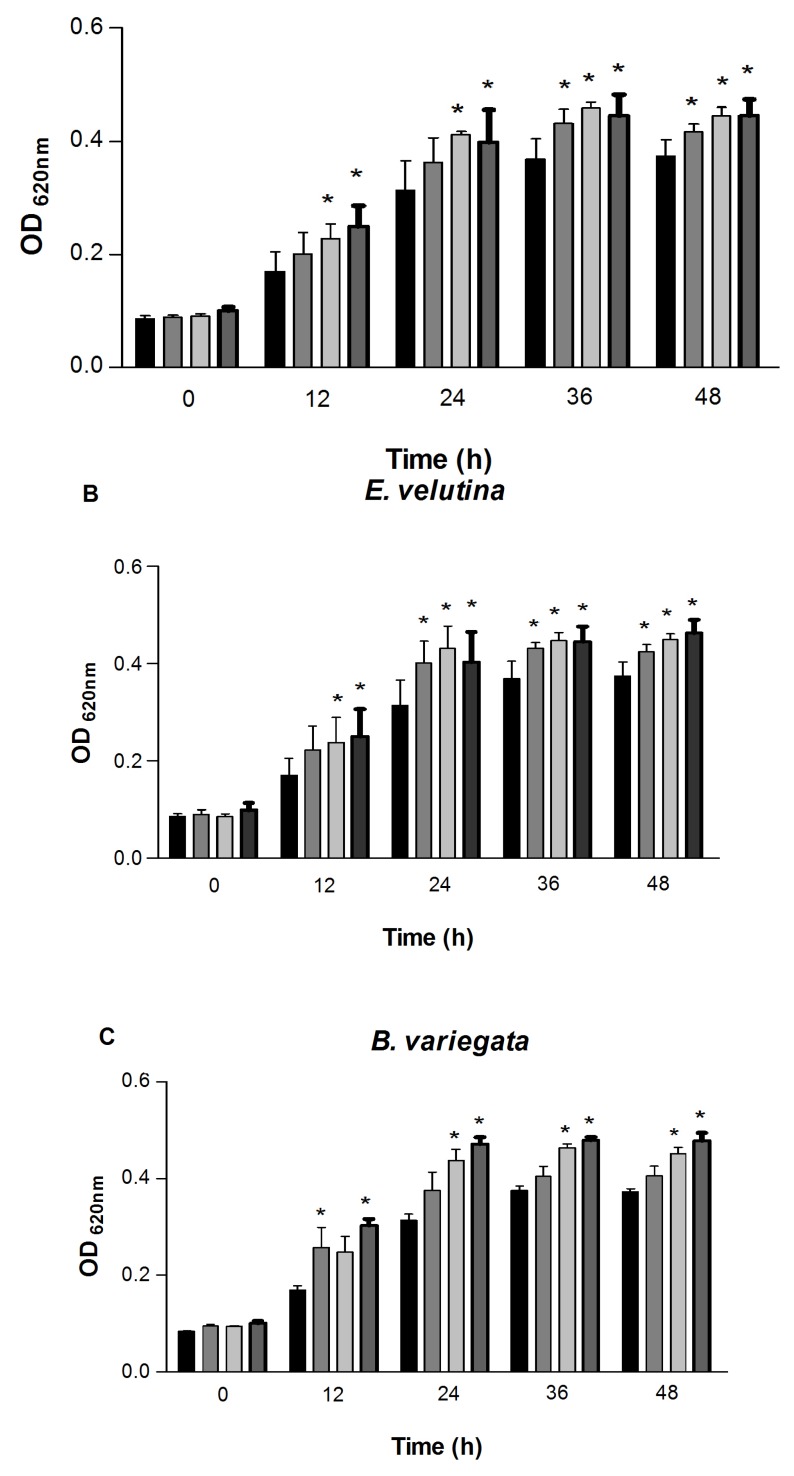
Effect of d-galactose-specific lectins on the growth of *R. tropici*. (**A**) VML, (**B**) EVL and (**C**) BVL. * *p* < 0.01 relative to control. Error bars indicate the standard deviation of the means. (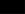) Control, (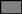) lectins at 125 µg/mL, (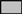) 250 µg/mL and (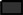) 500 µg/mL.

The effect of the lectins on CIAT899 growth was detected at concentrations of 125, 250 and 500 µg/mL and was monitored by measuring the optical density at 620 nm every 12 h, during 48 h. The results are shown in the Figure 2 and Figure 3.

Neither glucose/mannose- nor galactose-specific lectins affected the growth of *R. tropici* CIAT899 in concentrations lower than 125 µg/mL and the non-lectin protein control bovine serum albumin (BSA) did not differ significantly from the negative control (data not shown).

According to the data analysis, it was observed that DRL at a concentration of 500 µg/mL was able to stimulate rhizobial growth after 12 h. The same effect was seen at 125, 250 and 500 µg/mL after 36 and 48 h. Interestingly, DRL had no activity after 24 h of growth (Figure 2A). DML stimulated growth at concentrations 125, 250 and 500 µg/mL for all tested times (Figure 2B). DVL was effective at the same concentrations, but only after 36 and 48 h (Figure 2C). 

Despite the high degree of similarity shared by the d-glucose/d-mannose-binding lectins DML, DRL and DVL, DRL stimulated the growth of *R. tropici* more efficiently than all other proteins tested. Among the galactose-specific lectins, VML was able to stimulate rhizobial growth at concentrations of 250 and 500 µg/mL after 12 and 24 h and at 125, 250 and 500 µg/mL after 36 and 48 h (Figure 3A). 

EVL stimulated growth at 250 and 500 µg/mL after 12 h. The same effect was seen at 125, 250 and 500 µg/mL after 24, 36 and 48 h (Figure 3B). Similar to galactose-specific lectins, BVL was also able to stimulate rhizobial growth at 250 and 500 µg/mL after 24, 36 and 48 h. Interestingly, BVL stimulated rhizobial growth only at 125 and 500 µg/mL after 12 h and was not effective at the concentration of 250 µg/mL (Figure 3C). 

The present study evaluates the capacity of the leguminous lectins to stimulate the growth of *R. tropici* CIAT 899 *in vitro*. All lectins tested were able to significantly increase rhizobial growth, but with different levels of stimulus (Figure 2 and Figure 3). These results agree with those of previous studies [17,18] that reported the effects of interaction and stimulation on the growth of *R. leguminosarum* and *R. tropici* by *Pisum sativum* and *Canavalia brasiliensis* (ConBr) lectins, respectively. Vasconcelos *et al.* [18] presented the most recent findings on ConBr, a d-glucose/d-mannose-specific lectin, showing that its effects on the interaction and stimulus of rhizobial growth in the presence of 0.1 M of d-mannose were abruptly inhibited, clearly revealing that carbohydrate binding activity is essential for the stimulation of bacterial growth.

The diocleinae lectins used in this study share a high degree of similarity and bind to d-glucose and d-mannose [21]. Despite their high similarity, these lectins induced different biological activities in different assays, as well as nitric oxide production [22], anti-inflammatory effect [23] and inhibition of biofilm formation [24]. Moreover, Vasconcelos *et al.* [18] have shown that concanavalin A (ConA), unlike ConBr, was unable to stimulate *R. tropici* growth, even though both lectins could bind to rhizobial cells. According to Cavada *et al.* [21], small differences in the relative orientation of the carbohydrate-binding sites or pH-dependent oligomerization state may explain the differences of activities exerted by these lectins.

Previous studies with VML showed that this lectin was able to stimulate the metabolism of *R. tropici* by increasing the efflux of H^+^ [15], as well as stimulating the respiration of *R. tropici* and *R. etli* [16]. The mechanisms underlying the effect of these lectins on rhizobial growth remain unclear. Nevertheless, since VML stimulates the metabolism of *R. tropici*, it is plausible that the lectins used in the present study may be able to stimulate the cellular metabolism of *R. tropici* in a similar manner.

Lectins are able to recognize carbohydrates on the cell surface. Such molecules can also interact with cell wall polysaccharides and/or glycoconjugates in the cell membrane [25]. Laus and colleagues [26] demonstrated that 95% of all polysaccharides found on the surface of a *Rhizobium leguminosarum* strain are constituted from mannose and glucose, with minor amounts of galactose and rhamnose. Therefore, the effect of tested lectins may be related to the interaction between lectins and carbohydrate residues located on the surface of *R. tropici*. Nevertheless, some studies have shown the importance of lipopolysaccharide (LPS) on successful conclusion of the whole symbiotic process [27,28]. In addition to the lipid portion, the LPS structure consists of a saccharide portion, which is divided in an oligosaccharide (core) and an O-specific polysaccharide (OPS) [29]. The core region of some *Rhizobium* strains has a common structure consisting of an octasaccharide containing one residue of mannose, two of galactose, three of galacturonic acid and three of 3-deoxy-d-manno-2-octulosonic acid [30]. Such residues can act as anchor points for the lectins tested in this study. Thus, it is plausible to believe that the effect in the growth of *R. tropici* was exerted by the lectins that interacted with specific residues located on the surface of this bacterium.

## 3. Experimental

### 3.1. Lectin Purification

Lectins from *D. violacea*, *D. megacarpa*, *D. rostrata*, *V. macrocarpa*, *E. velutina* and *B. variegata* were isolated from seeds as previously described [31,32,33,34,35,36], with minor modifications. The purification steps are briefly described below:

#### 3.1.1. *Dioclea* Lectin Purification

The seeds of *D. megacarpa*, *D. rostrata* and *D. violacea* were separately ground into a fine powder by a coffee mill. The powder was stirred with 0.15 M NaCl (1:10 w/v) at room temperature for 4 h. The extract was centrifuged at 20,000 g for 20 min at 4 °C and the supernatant of seeds was applied to a Sephadex G-50 column (Amersham Biosciences, Salt Lake City, UT, USA) pre-equilibrated with 0.15 M NaCl containing 5 mM CaCl_2_ and 5 mM MnCl_2_. After removing the unbound material, each lectin was eluted with 0.1 M of glucose in equilibration solution. Then, this fraction was submitted to a 1 h dialysis against 0.1 M acetic acid, followed by extensive dialysis dialyzed against distilled water. Finally, the lectins were freeze-dried and stored at 4 °C for later use.

#### 3.1.2. VML Purification

The seeds of *V. macrocarpa* were ground into a fine powder by a coffee mill, defatted with *n*-hexane and air-dried at room temperature. The powder was stirred with 0.15 M NaCl (1:10 w/v) at room temperature for 4 h. The extract was centrifuged at 20,000 g for 20 min at 4 °C, and the resulting clear supernatant was applied to a Guar gum (Sigma-Aldrich, St. Louis, MO, USA) column equilibrated with 0.15 M NaCl. After washing out the unbound material, the lectin was eluted with 0.1 M d-galactose in 0.15 M NaCl and this fraction was submitted to a 1 h dialysis against 0.1 M acetic acid, followed by and extensive dialysis against distilled water. Finally, the pure lectin was freeze-dried and stored at 4 °C for future use.

#### 3.1.3. BVL Purification

Ground seeds of *B. variegata* were homogenized with 0.1 M Tris-HCl, pH 7.6, containing 0.15 M NaCl (1:20 w/v) for 4 h at room temperature. After centrifugation at 20,000 g for 20 min at 4 °C, the supernatant containing the protein was loaded onto a lactose–agarose (Sigma) column equilibrated and eluted with 0.1 M Tris-HCl, pH 7.6, containing 0.15 M NaCl. Bound proteins were eluted with 0.1 M lactose in equilibration buffer. The fractions were collected, pooled and dialyzed against 0.1 M acetic acid, followed by extensive dialysis against distilled water. The lectin was then aliquoted, freeze-dried and stored for future use.

#### 3.1.4. EVL Purification

The seeds of *E. velutina* were ground into a fine powder by a coffee mill. The powder was stirred with 0.1 M Tris-HCl, pH 7.6, containing 0.15 M NaCl, for 4 h at room temperature. After centrifugation at 20,000 g for 20 min at 4 °C the supernatant was extensively dialyzed against water and centrifuged to provide a precipitated and soluble fraction. The fraction was further fractionated by the addition of ammonium sulfate. The material precipitated between 30–90% of saturation was recovered and dialyzed in 0.15 M NaCl. The precipitate was retired and the supernatant applied to the Guar gum column (Sigma-Aldrich) also equilibrated also with 0.15 M NaCl. After washing out the unbound material, the lectin was eluted with 0.1 M d-galactose in 0.15 M NaCl. This fraction was submitted to a 1 h dialysis against 0.1 M acetic acid, followed by extensive dialysis against distilled water. The lectin was then freeze-dried and stored at 4 °C for future use.

### 3.2. SDS-PAGE

The purity of the lectins was assessed by SDS-PAGE as described by Laemmili [37]. Samples of each protein were submitted to electrophoresis using 15% polyacrylamide gel. Proteins were boiled in loading buffer (50 mM Tris-HCl, pH 6.8, 2% SDS, 0.1% bromophenol blue (3',3",5',5"-tetrabromophenolsulfonphthalein, BPB, albutest, 10% glycerin) for 5 min and an aliquot was loaded in each lane. High range protein standards (GE Healthcare, Salt Lake City, UT, USA,) were used as molecular weight markers.

### 3.3. Cultivation of Microorganisms

The rhizobial strain CIAT899 was stock-cultured on yeast-mannitol (YM) broth [38] containing 20% glycerol and stored at −80 °C. The inoculum was then transferred to Petri dishes containing YM agar and incubated at 28 °C for 48 h. Subsequently, a colony was carefully transferred to 10 mL YM broth and incubated at 28 °C for 48 h under constant agitation (120 rpm). Immediately before use, the bacterial suspension was adjusted to 1 × 10^8^ cells/mL using McFarland standards.

### 3.4. Bacterial Growth Assay

The bacterial growth assay was carried out as previously described by Vasconcelos *et al.* [18]. The assay was performed using 96-well polystyrene plates. The pure lectins were dissolved in 0.15 M NaCl, then filtered through 0.22 µm filters and serially diluted in 0.15 M NaCl from 500 to 31.25 µg/mL. Then, 100 µL of each lectin dilution was added to a new plate, plus 100 µL of bacterial suspension in YM (1 × 10^8^ cells/mL), and incubated at 28 °C for 12 h in an orbital shaker at 120 rpm. Afterwards, 20 µL of the previously incubated suspension was inoculated in 180 µL YM broth and incubated again at 28 °C at 120 rpm. The optical density at 620 nm (OD 620nm) was measured at 12, 24, 36 and 48 h using a microplate reader (Biotrak II Visible Plate ReaderAmersham Biosciences). Bovine serum albumin (BSA, Sigma-Aldrich) was used as a non-lectin protein control and 0.15 M NaCl + YM broth as a negative control. 

### 3.5. Statistical Analyses

Statistical analyses were performed by GraphPad Prism^®^ version 5.0 for Microsoft Windows^®^. The data from 3 different assays were compared using one-way analysis of variance (ANOVA), with Bonferroni post-hoc test. The data were considered statistically significant when *p* < 0.01. 

## 4. Conclusions

In conclusion, this study shows that both d-galactose- and also d-glucose/d-mannose-binding lectins purified from seeds of leguminous plants may be powerful biotechnological tools to stimulate the growth of *R. tropici* CIAT899, thus symbiotic interaction between rhizobia and the common bean and, hence, the production of this important field crop. Although the mechanisms underlying the stimulus of bacterial growth remain unclear, it can be plausibly speculated that act by modulating the metabolism of *R. tropici* CIAT899 to increase its growth. However, further studies are necessary to properly elucidate the mechanisms of action involved in lectin stimulus of rhizobial growth. 
